# The Association of Radiation Therapy and Chemotherapy on Overall Survival in Merkel Cell Carcinoma: A Population-Based Analysis

**DOI:** 10.7759/cureus.18276

**Published:** 2021-09-25

**Authors:** Aleksander Vayntraub, Nadine Tayeb, Bryan Squires, Janice M Mehnert, Quais Hassan II, Nikhil T Sebastian, Rohan Deryaniyagala, Thomas J Quinn

**Affiliations:** 1 Department of Radiation Oncology, Beaumont Health, Royal Oak, USA; 2 Department of Radiation Oncology, Michigan State University College of Human Medicine, East Lansing, USA; 3 Laura and Isaac Perlmutter Cancer Center, New York University Langone Medical Center, New York, USA; 4 Medical Scientist Training Program, The Ohio State University College of Medicine, Columbus, USA; 5 Department of Radiation Oncology, Winship Cancer Institute of Emory University, Atlanta, USA

**Keywords:** chemotherapy, radiation therapy, overall survival, radiotherapy, chemo radiotherapy (chemo-rt), merkel cell carcinoma

## Abstract

Purpose/objective(s)

Merkel cell carcinoma (MCC) is a rare, aggressive cutaneous neoplasm traditionally managed with surgical resection followed by radiotherapy (RT). With the recent approval of checkpoint inhibitors, chemotherapy is less commonly utilized. We analyzed the impact of RT and chemotherapy on overall survival (OS) in patients with MCC using Surveillance, Epidemiology, and End Results (SEER), a population-level database.

Materials and methods

We performed retrospective analyses on SEER 18 Custom Data registries for MCC (ICD-0-3 8247). Data from 1980 to 2016 was queried for analysis, and an initial list of 9,792 patients was populated (ICD: C00, C07.9, C44, C80.9). Selection for cases with chemotherapy and RT status, single primary tumor, primary tumor location and surgery treatment type yielded 5,002 cases for analysis. Baseline characteristics were compared with Chi-square or Mann-Whitney U test. Univariate and multivariable analysis using Kaplan-Meier and Cox proportional hazards regression modeling were performed. Propensity-score matched analysis with inverse probability of treatment weighting (IPTW) was used to account for indication bias.

Results

Median follow-up time was 178 months (68 to 217 months). Independent prognostic factors positively correlated with increased OS, for both unadjusted Multivariate analysis and IPTW adjusted MVA were age, male sex, year of diagnosis, stage, RT status, and chemotherapy status. On adjusted MVA, use of chemotherapy was associated with worse OS (hazard ratio: 1.22 [95% CI 1.1-1.35], p<0.001), whereas RT was associated with improved OS (HR:0.9 [95% CI, 0.83-0.97], p=0.008).

Conclusions

The current study demonstrates that RT is associated with improved survival for patients with MCC. Chemotherapy was associated with worse OS. This supports the recent clinical shift towards immune checkpoints inhibitors as standard of care in the metastatic setting, and promising trials in the adjuvant and advanced settings.

## Introduction

Merkel cell carcinoma (MCC) is an aggressive cutaneous malignancy of neuroendocrine origin. The incidence in the United States is rare: approximately 1,500 cases were diagnosed in 2007 with a projected increased annual incidence to 3,284 cases in 2025 [1]. MCC has high metastatic potential, and many patients develop recurrent disease. In a modern study by Fields et al., 108 of 364 patients (29.7%) with Stage I-III MCC who underwent complete resection experienced recurrences at local (10%), in-transit (11%), nodal (40%), and distant (39%) sites [2], and most recurrences occurred within two years. Higher recurrence rates are associated with advanced stage, age > 70, tumor size > 2 cm, positive lymph node status, lymphovascular stromal invasion (LVSI), and male sex [3,4].

Historically, surgical resection with wide margins has been the mainstay of therapy. However, given the high rates of locoregional relapse (LRR) and radiosensitivity of MCC, radiation therapy (RT) has been increasingly utilized as an adjuvant therapy since the 1980s [3]. In contrast, the current role for systemic therapy is limited and historically included cytotoxic chemotherapy but more recently, immunotherapy has become standard of care in the advanced and metastatic disease setting [5-8]. Adjuvant chemotherapy is not currently recommended, though clinical trial participation is encouraged [9].

Given the rarity of MCC and subsequent lack of Phase III clinical trials for RT and chemotherapy, retrospective analyses of population-based data, such as the Surveillance, Epidemiology, and End Results (SEER) database, offer an avenue to inform treatment and future studies. Most RT series and database analyses have supported the role of RT in the management of MCC. A SEER analysis of 1,665 MCC cases by Mojica et al. in 2007 demonstrated that the addition of RT to MCC treatment provided a median survival benefit of 18 months and noted a benefit for patients with lesions > 2 cm [10]. In the metastatic setting, immunotherapy has come to the forefront of standard of care with objective response rates (ORR) up to 68%6 and long durable response [11]. Although MCC shows objective responses to chemotherapy regimens, these responses are not durable [8]. Chemotherapy efficacy is further limited by toxicity, particularly among the frail. Retrospective studies have reported a response rate of ~ 55% in the setting of metastatic disease, while non-metastatic studies show varied response rates [9].

As the role for chemotherapy has not yet been analyzed in the SEER registry, the goal of the current study is to further evaluate and characterize the association between the use of chemotherapy and radiation therapy on overall survival (OS) in patients with stage I-IV MCC. Additionally, we evaluate other variables for MCC for their prognostic significance.

## Materials and methods

Data source

The Surveillance, Epidemiology, and End Results (SEER) Program (v8.3.6, The Surveillance Research Program of the Division of Cancer Control and Population Sciences, National Cancer Institute) collects and publishes cancer incidence and survival data from population-based cancer registries covering approximately 34% of the US population. We used the specialized Radiation/Chemotherapy Database (SEER 18 Custom Data, November 2018 Submission) as it contains information on RT and chemotherapy. 

Cohort analyzed

**Figure 1:**
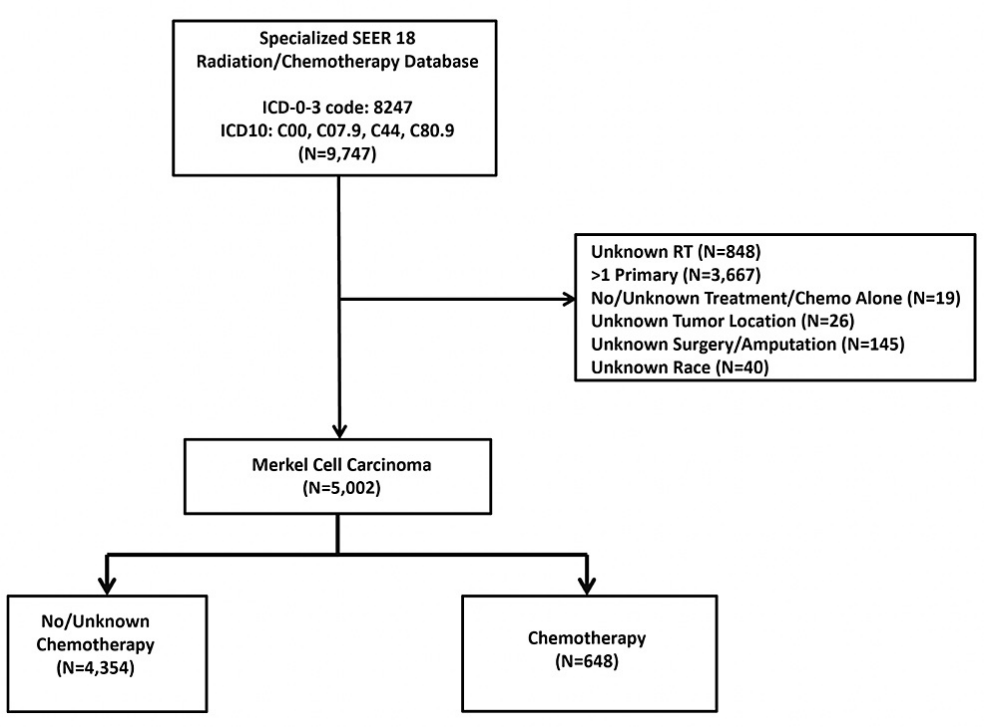
CONSORT diagram of selection criteria for Merkel cell carcinoma (MCC) cases in the SEER 18 population-based cancer database. From 9,747 total database entries, 5,002 cases of MCC without exclusion criteria were identified and evaluated further. SEER: Surveillance, Epidemiology, and End Results; CONSORT: Consolidated Standards Of Reporting Trials.

From 1980 to 2016, the SEER 18 Custom database was queried for a diagnosis of Merkel Cell Carcinoma corresponding to International Classification of Disease for Oncology (ICD-0-3) code 8247 and topographical codes (ICD: C00, C07.9, C44, C80.9). Inclusion criteria were cases with indicated chemotherapy and RT status, single primary tumor, known primary tumor location, and known surgery treatment type. Exclusion criteria included primary anatomic sites other than “Trunk”, “Upper Extremity”, “Lower Extremity”, “Head and Neck”, and “Skin, Not otherwise specified (NOS)”. A total of 5,002 patients with MCC were included in the final analysis (Figure 1).

Statistical analysis

Baseline patient characteristics were assessed, before and after propensity score (PS) matching with inverse probability of treatment weighting (IPTW), using χ2 and standard mean difference (SMD), where a SMD > 0.1 was considered imbalanced [12]. Univariate analysis (UVA) of patient characteristics’ impact on overall survival (OS) was performed using the Kaplan-Meier (KM) method, with the log-rank method to assess for significance. Multivariate analysis (MVA) of patient characteristics and OS was performed using Cox proportional hazards regression modeling. Covariates with p < 0.1 in the UVA were incorporated in multivariate Cox proportional hazards regression modeling using backward stepwise methodology to mitigate collinearity of variables and overfitting of the final MVA model. These methods were performed as described in our previous work [13].

A PS-matched analysis was performed to account for indication bias. Propensity scores were estimated using binary logistic regression modeling for receipt of no/unknown chemotherapy or chemotherapy [13]. Next, IPTW were calculated as 1/PS and 1/(1-PS) [14]. Stabilization of the IPTWs was performed by multiplying the standard IPTWs by the probability of undergoing treatment that each patient received [15]. Finally, IPTW-adjusted UVA and doubly robust, IPTW-adjusted MVA were performed as described previously [16].

All statistical tests were completed using SEER*Stat (v8.3.5, The Surveillance Research Program of the Division of Cancer Control and Population Sciences, National Cancer Institute) and R version 3.6.2 statistical software (R Foundation for Statistical Computing, Vienna, Austria). Furthermore, all statistical analyses were performed as two-sided with p < 0.05 considered statistically significant. R markdown for all analyses is available upon request.

## Results

Patient demographics

MCC patient baseline and treatment characteristics in the SEER 18 dataset were tabulated (Table 1). The median age at diagnosis of MCC was 76 years. Median follow-up time was 14 years and 10 months. This cohort of patients was comprised mostly of those in the 60-79 years of age category (48%) and 80+ years of age (39%), with the remainder in the < 60 years age group (13%). Moreover, the vast majority of patients were diagnosed in 2000 or later, with “2000-2009” comprising 45%, and “2010 - 2016” comprising 42% of MCC diagnoses. Insurance status was “insured” for 51%, or “unknown” for 46% of cases, whereas “Medicaid” and “uninsured” comprised 2.8% and 0.4%, respectively. The group distribution based on sex was predominantly male (59%).

**Table 1:** SEER 18 Merkel cell carcinoma dataset characteristics before and after IPTW-adjustment and stratification by chemotherapy status. ^1^Statistics presented: median (IQR); n (%). IPTW: inverse probability of treatment weighting; SEER: Surveillance, Epidemiology, and End Results.

Characteristics^1^	N = 5002	IPTW-adjusted			
Chemotherapy Status			Chemotherapy		
No/Unknown	4354 (87%)		No/Unknown	Yes	p
Yes	648 (13%)		N=4387.9	N=624.3	
Age at Diagnosis (months)	76 (66, 83)	Age at Diagnosis (median [IQR])	76 (667, 83)	76 (67, 83)	0.97
Age Category		Age Category			0.221
<60	663 (13%)	<60	589.1 (13.4)	67.3 (10.8)	
60-79	2397 (48%)	60-79	2101.6 (47.9)	283.6 (45.4)	
80+	1942 (39%)	80+	1697.1 (38.7)	273.4 (43.8)	
Insurance Status		Insurance Status			0.575
Insured	2555 (51%)	Insured	2221.7 (50.6)	292.9 (46.9)	
Medicaid	140 (2.8%)	Medicaid	124.3 ( 2.8)	16.7 ( 2.7)	
Uninsured	21 (0.4%)	Uninsured	17.4 ( 0.4)	3.9 ( 0.6)	
Unknown	2286 (46%)	Unknown	2024.4 (46.1)	310.7 (49.8)	
Sex		Sex			0.727
Female	2068 (41%)	Female	1795.7 (40.9)	264.4 (42.3)	
Male	2934 (59%)	Male	2592.2 (59.1)	359.9 (57.7)	
Year of Diagnosis		Year of Diagnosis			0.198
1980-1999	637 (13%)	1980-1999	574 (13.1)	82.1 (13.2)	
2000-2009	2244 (45%)	2000-2009	1966.5 (44.8)	316 (50.6)	
2010-2016	2121 (42%)	2010-2016	1847.3 (42.1)	226.1 (36.2)	
Follow-up Time (months)	178 (68, 217)	Follow-up Time (median [IQR])	178 (68.52, 217)	162.53 (69, 215)	0.51
Tumor Location		Primary Site			0.948
Trunk	489 (9.8%)	Trunk	430.7 ( 9.8)	61.6 ( 9.9)	
Head and Neck	2201 (44%)	Head and Neck	1918.7 (43.7)	276.7 (44.3)	
Lower Extremity	791 (16%)	Lower Extremity	687.4 (15.7)	103.4 (16.6)	
Skin, NOS	257 (5.1%)	Skin, NOS	254.9 ( 5.8)	39.3 ( 6.3)	
Upper Extremity	1264 (25%)	Upper Extremity	1096.3 (25)	143.3 (22.9)	
Tumor Grade		Grade			0.977
I	12 (0.2%)	I	9.3 ( 0.2)	0.7 ( 0.1)	
II	13 (0.3%)	II	11.4 ( 0.3)	1.6 ( 0.3)	
III	455 (9.1%)	III	403.1 ( 9.2)	54.4 ( 8.7)	
IV	285 (5.7%)	IV	260.9 ( 5.9)	37.4 ( 6)	
Unknown	4237 (85%)	Unknown	3703.2 (84.4)	530.2 (84.9)	
SEER Summary Stage		SEER Summary Stage			0.832
Localized	2226 (45%)	Localized	1936.2 (44.1)	283.9 (45.5)	
Regional	1752 (35%)	Regional	1524.6 (34.7)	211.5 (33.9)	
Distant	390 (7.8%)	Distant	376.7 ( 8.6)	58.5 ( 9.4)	
Unknown	634 (13%)	Unknown	550.5 (12.5)	70.3 (11.3)	
Type of Surgery		Surgery			0.278
Biopsy/Local Destruction	1119 (22%)	Biopsy/Local Destruction	973.5 (22.2)	153.2 (24.5)	
Local Excision	1476 (30%)	Local Excision	1279.8 (29.2)	147.5 (23.6)	
Wide Local Excision	1931 (39%)	Wide Local Excision	1684.6 (38.4)	265.1 (42.5)	
No Definitive Surgery	476 (9.5%)	No Definitive Surgery	449.9 (10.3)	58.4 ( 9.4)	
Radiation Therapy		Radiation Therapy			0.306
No	2452 (49%)	No	2128.9 (48.5)	276.5 (44.3)	
Yes	2550 (51%)	Yes	2259 (51.5)	347.8 (55.7)	
Vital Status		Marital Status			0.62
Alive	2104 (42%)	Single	342.1 ( 7.8)	33 ( 5.3)	
Dead	2898 (58%)	Married/Domestic Partner	2497.1 (56.9)	349.1 (55.9)	
		Divorced/Separated	292.3 ( 6.7)	37.5 ( 6)	
		Widowed	913.1 (20.8)	147.8 (23.7)	
		Unknown	343.2 ( 7.8)	56.9 ( 9.1)	
		Race			0.456
		White	4195.1 (95.6)	603.6 (96.7)	
		Black	59 (1.3)	5 (0.8)	
		Other	133.7 ( 3)	15.7 ( 2.5)	
		Laterality			0.269
		Bilateral	7.9 ( 0.2)	0.9 ( 0.2)	
		Unilateral	3764.7 (85.8)	514.5 (82.4)	
		Unpaired	615.3 (14)	108.9 (17.4)	

Tumor characteristics

MCC tumor characteristics in the SEER 18 dataset included the following anatomic distribution: Trunk (9.8%), Head and Neck (44%), Lower Extremity (16.8%), Upper Extremity (25%) and Skin, not-otherwise-specified (NOS) (5.1%). Histologic grade was unknown in most cases (85%). The remaining data was separated by Grade 1 (0.2%), Grade 2 (0.3%), Grade 3 (9.1%) and Grade 4 (de-differentiated) at 5.7% of the dataset. Most patients presented with locoregionally confined disease with 45% categorized as “localized”, and 35% “regional”; only 7.8% had distant metastases at diagnosis while the remaining 13% were staged as “unknown”. Slightly more than half (51%) of patients received RT. Chemotherapy administration was reported in 13% of cases. Most patients underwent definitive surgical resection defined as “local excision” (30%) and “wide local excision” (49%). In contrast, only 22% of patients underwent “biopsy/local destruction” and 9.5% were categorized as having “no surgery”. Following PS-matching and IPTW, all baseline patient and tumor characteristics assessed were similar, regardless of receipt of chemotherapy (Table 2).

**Table 2:** Univariate and multivariate weighted analysis of SEER 18 MCC dataset. HR: hazard ratio; CI: confidence interval; IPTW: inverse probability of treatment weighting; NOS: not-otherwise-specified; UVA: univariate analysis; MVA: multivariate analysis; MCC: Merkel cell carcinoma.

Characteristic	IPTW Adjusted UVA			IPTW Adjusted MVA		
	HR	95% CI	p-value	HR	95% CI	p-value
Age Category						
<60	—	—		—	—	
60-79	1.43	1.23, 1.66	<0.001	1.39	1.19, 1.61	<0.001
80+	2.04	1.76, 2.36	<0.001	1.86	1.60, 2.17	<0.001
Race						
White	—	—		—	—	
Black	0.9	0.64, 1.27	0.5	0.96	0.68, 1.36	0.8
Other	0.83	0.65, 1.05	0.12	0.82	0.64, 1.04	0.1
Insurance Status						
Insured	—	—		—	—	
Medicaid	1.19	0.92, 1.55	0.2	1.29	0.99, 1.69	0.06
Uninsured	0.94	0.49, 1.79	0.8	0.89	0.47, 1.71	0.7
Unknown	2.36	2.19, 2.55	<0.001	1.91	1.71, 2.13	<0.001
Sex						
Female	—	—		—	—	
Male	1.24	1.16, 1.34	<0.001	1.34	1.24, 1.44	<0.001
Year of Diagnosis						
1980-1999	—	—		—	—	
2000-2009	0.72	0.66, 0.79	<0.001	0.84	0.76, 0.93	<0.001
2010-2016	0.38	0.34, 0.42	<0.001	0.76	0.66, 0.88	<0.001
Primary Site						
Trunk	—	—		—	—	
Head and Neck	1.01	0.89, 1.14	>0.9	1.05	0.93, 1.20	0.4
Lower Extremity	0.93	0.81, 1.08	0.4	1.03	0.89, 1.20	0.7
Skin, NOS	0.96	0.79, 1.16	0.7	1.14	0.92, 1.41	0.2
Upper Extremity	0.8	0.70, 0.92	0.002	0.93	0.80, 1.07	0.3
SEER Summary Stage						
Localized	—	—		—	—	
Regional	1.04	0.96, 1.12	0.4	1.15	1.06, 1.26	<0.001
Distant	1.47	1.31, 1.65	<0.001	1.48	1.30, 1.68	<0.001
Unknown	0.53	0.46, 0.62	<0.001	0.64	0.55, 0.75	<0.001
Surgery						
Biopsy/Local Destruction	—	—		—	—	
Local Excision	0.72	0.65, 0.79	<0.001	0.92	0.83, 1.02	0.13
Wide Local Excision	0.92	0.84, 1.01	0.081	1.01	0.92, 1.11	0.8
No Definitive Surgery	0.94	0.82, 1.07	0.3	0.98	0.84, 1.15	0.8
Radiation Therapy						
No	—	—		—	—	
Yes	0.85	0.79, 0.91	<0.001	0.9	0.83, 0.97	0.008
Chemotherapy						
No/Unknown	—	—		—	—	
Yes	1.3	1.18, 1.44	<0.001	1.22	1.10, 1.35	<0.001
Grade						
I	—	—				
II	1.35	0.42, 4.31	0.6			
III	1.65	0.65, 4.16	0.3			
IV	1.95	0.77, 4.94	0.2			
Unknown	1.42	0.57, 3.57	0.5			
Laterality						
Bilateral	—	—				
Unilateral	0.53	0.25, 1.14	0.1			
Unpaired	0.65	0.30, 1.41	0.3			
Marital Status						
Single	—	—				
Married/Domestic Partner	0.96	0.83, 1.11	0.6			
Divorced/Separated	0.97	0.79, 1.19	0.7			
Widowed	1.12	0.96, 1.31	0.14			
Unknown	1.08	0.89, 1.30	0.4			

Univariate analysis

**Figure 2:**
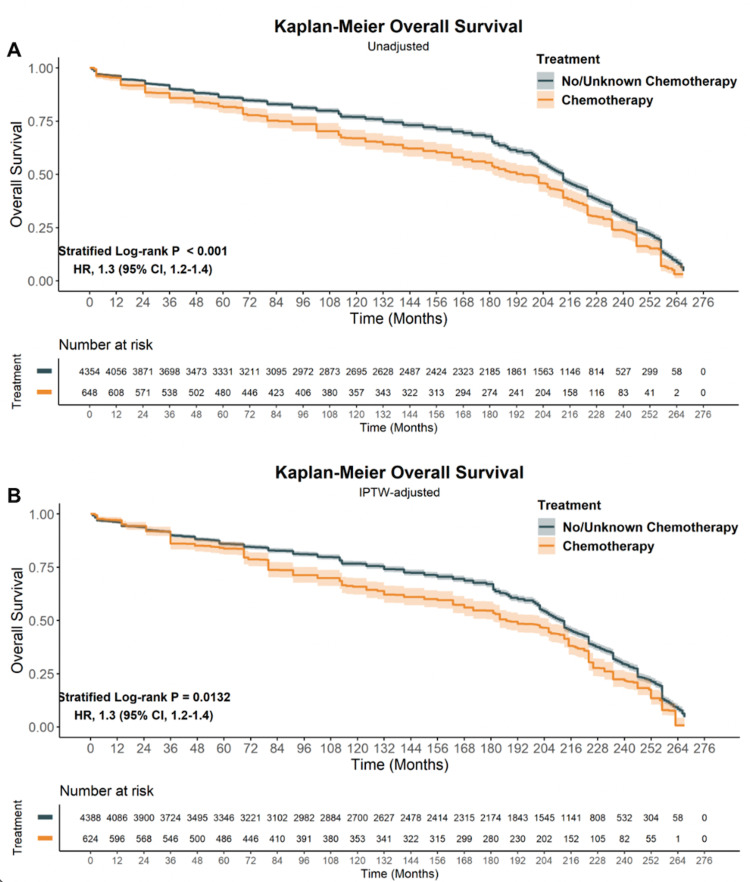
Overall survival (OS) for MCC in SEER 18 stratified by chemotherapy status. A: OS derived from raw unadjusted data. B: OS after IPTW adjustment. HR: hazard ratio; CI: confidence interval; IPTW: inverse probability of treatment weighting; NOS: not-otherwise-specified; UVA: univariate analysis; MVA: multivariate analysis; MCC: Merkel cell carcinoma; OS: overall survival.

The impact of patient, tumor, and treatment characteristics on OS were evaluated using the Kaplan-Meier method (KM) [14]. Table 2 provides the UVAs for the unadjusted and PS-matched IPTW-adjusted no/unknown and yes chemotherapy. In the unadjusted population, older age at diagnosis, male sex, distant disease, and receipt of chemotherapy (Figure 2A. HR 1.31 [95% CI, 1.18-1.45] p < 0.001) were poor prognostic features. Conversely, later calendar year of diagnosis, upper extremity location, definitive surgical resection, and receipt of RT (HR 0.84 [95% CI 0.78-0.9] p < 0.001) were protective. Following PS-matching and IPTW, the aforementioned factors remained prognostic (Table 2).

Multivariable analysis

On doubly robust MVA (Table 2), the Hazard Ratio for death was both worse and statistically significant for the following factors: age 60-79 (HR 1.39 [95% CI, 1.19-1.61] p < 0.001), age 80+ (HR 1.86 [95% CI, 1.60-2.17], p < 0.001), male sex (HR 1.34 [95% CI 1.24-1.44], p < 0.001), regional disease (HR 1.15 [95% CI, 1.06-1.26], p < 0.001) and distant disease (HR 1.48 [95% CI, 1.30-1.68], p < 0.001), and receipt of chemotherapy (HR 1.22 [95% CI 1.10-1.35] p < 0.001). In contrast, year of diagnosis 2000-2009 (HR 0.84 [95% CI 0.76-0.93] p < 0.001); year of diagnosis 2010-2016 (HR 0.76 [95% CI, 0.66-0.88] p < 0.001) and use of radiation therapy (HR 0.9 [95% CI 0.83-0.97], p = 0.008) were associated with improved OS. No impact on OS was found based on anatomic location, race, insurance status, or surgery type. 

Exploratory subgroup analysis

**Figure 3:**
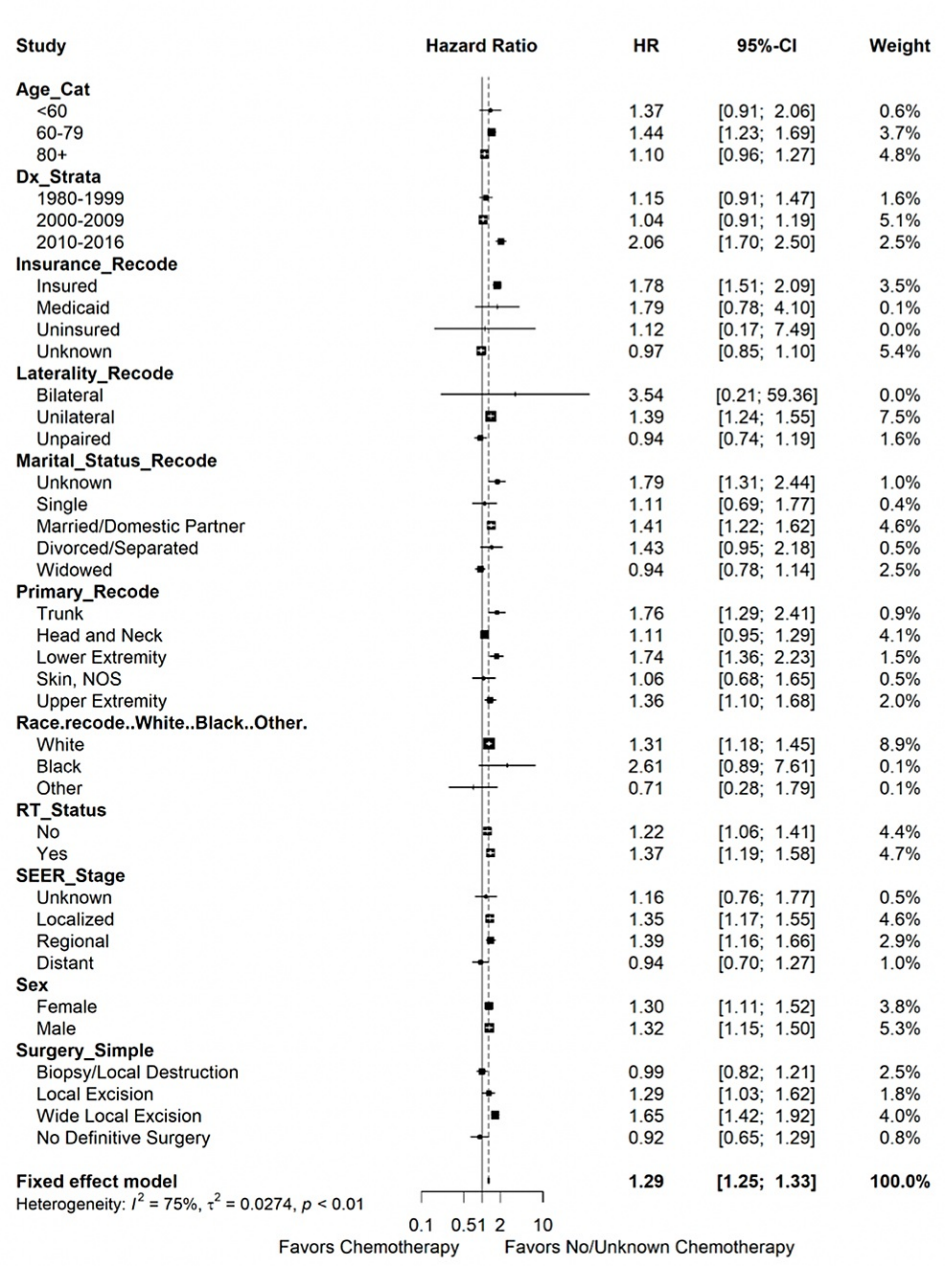
Forest plot describing the estimated effect of the interaction of chemotherapy with other prognostic factors on MCC overall survival.

To explore possible subgroups of MCC which may benefit from chemotherapy, we conducted a series of subgroup analyses reported as a Forest plot (Figure 3). Subgroups included age of diagnosis, calendar year of diagnosis, insurance type, laterality, marital status, anatomic subsite, race, RT status, stage, and surgery type. For all of the subgroups analyzed, there did not appear to be any OS improvement with the addition of chemotherapy. However, for the highest risk patients (80+ years old, distant metastatic disease, and no definitive surgery), chemotherapy was not associated with worse OS.

## Discussion

MCC remains a rare aggressive cutaneous neoplasm with a high propensity for recurrence and distant metastasis. Given its rarity, randomized trials for MCC are difficult to accrue. Large-database retrospective analysis can still help guide clinical management and inform investigation of future treatments. Not surprisingly, our analysis shows worse outcomes are correlated with increasing age and earlier calendar decade of diagnosis, as these are surrogates for poorer general health and older staging and treatment techniques. Male sex was also found to be a risk factor in our study, matching prior reports [3]. Retrospective analyses, series, and database studies comprise most of the available data guiding adjuvant management for MCC. Following optimal surgical excision for non-metastatic disease, most studies document a benefit to the use of adjuvant RT [9].

A multi-center retrospective study in 2011 by Ghadjar et al. looked at 180 patients with local or regional MCC treated between 1988 and 2009 in which surgery alone was compared with surgery and post-operative RT (n=131), surgery alone (n=49) or radical RT alone (n=13). With a median follow-up of 5 years, there was a significant benefit with the use of post-operative RT for LRFS (93% vs 64%), RRFS (76% vs 27%), DMFS (70% vs 42%), DFS (59% vs 4%) and CSS (65% vs 49%) in the radiotherapy group; however, there was no benefit to OS [17]. In a National Cancer Database (NCDB) study, a multi-variate analysis of 6,908 MCC patients found an OS benefit with the addition of adjuvant RT for patients with node-negative MCC compared with surgery alone (stage I: HR =0.71, 95% CI = 0.64 to 0.80, p < 0.001; stage II: HR = 0.77, 95% CI = 0.66 to 0.89, p < 0.001) , but not in those patients who were node positive (stage III: n = 2065) [18]. In a randomized trial by Jouary et al. evaluating the role of regional nodal irradiation, following wide local excision stage I MCC patients were randomized to adjuvant nodal and local tumor bed RT versus adjuvant local tumor bed RT alone. Accrual was stopped early. Although no improvements to OS were found for patients (n = 83) with a median follow-up of 57.7 months, there was a significant improvement in regional recurrence from 16% to 0% [19]. An analysis of the SEER registry by Mojica et al. in 2007 evaluated 1,667 cases with stage I-III MCC and demonstrated a survival benefit with the use of adjuvant radiotherapy. Median survival improved from 45 months for those who did not receive adjuvant irradiation to 63 months with adjuvant irradiation [10]. In contrast to the prior SEER study in 2007, our analysis studies the variable of chemotherapy use, included approximately three times as many patients, and included more patients treated in the modern era. Our results further support the use of RT in the management of MCC, as we noted a decreased risk of death (HR 0.9, [95% CI: 0.83 - 0.97] p = 0.008) on doubly robust MVA. Although sequence of therapy was unavailable in the SEER data, because 69% of the cohort had definitive local surgical therapy with an additional 22% having “biopsy/local destruction” this benefit is likely carried by the adjuvant RT cohort.

In contrast to the benefit we found with radiotherapy, our results indicate that chemotherapy is associated with worsened OS, with an increased risk of death (HR 1.3, p < 0.001) on doubly robust MVA. Only on subgroup analysis for the highest risk patients (80+ years old, distant metastatic disease, and no definitive surgery), chemotherapy was not associated with worse OS but also did not offer benefit for OS. This may be due to limited power within the subgroup. The lack of OS benefit with chemotherapy use coincides with the published literature, as studies report significant objective response rates, but short progression-free survival and no survival advantage [20-22]. Because of similar neuroendocrine origin, many of the traditional cytotoxic regimens used to treat MCC have been extrapolated from small cell carcinoma therapy. Treatments such as cyclophosphamide, doxorubicin and vincristine (CAV) or carboplatin and etoposide (EP) have reported response rates of 29% to 75% in the advanced and metastatic settings [9]. In a report on 107 MCC cases with locally advanced or metastatic disease, Voog et al. showed a reasonable objective response rate of 61% with first-line chemotherapy and 45% with second-line chemotherapy. But overall, there was no adequate cure rate using chemotherapy, and a high incidence of toxic death was reported (7.7% with first-line treatment) [23]. A more recent evaluation for patients with metastatic MCC by Becker et al. in 2017 demonstrated a low objective response rate of 8.8% with a median duration of 1.9 months with second-line or later chemotherapy [24]. Newer studies demonstrating immunotherapy efficacy have established a new standard of care with ORR up to 68%, durable responses [6] and upon interim analysis, suggestion that long-term OS may be possible even in the metastatic setting [11] - examples include immune checkpoint inhibitors such as avelumab, Nivolumab (Checkmate 358) [6], PD-1 inhibitors [25], and more unique therapies such as talimogene laherparepvec (TVEC) [9]. In the JAVELIN Merkel 200 phase II study, 88 patients receiving 10 mg/kg of avelumab by one-hour intravenous infusion every two weeks until progression, unacceptable toxicity, or disease response demonstrated a 33% objective response rate with 74% of responses lasting ≥ 1 year and median durable objective response not yet reached at the time of the updated analysis. One-year PFS and OS rates were 30% and 52%, respectively [11]. Although responses appeared more effective for those with fewer prior lines of systemic therapy, less burden of disease, and PD-L1 positive tumors, durable responses occurred in all subgroups irrespective of baseline factors. This led to FDA approval of avelumab in the treatment of metastatic MCC. Additionally, a recently published phase II trial (Cancer Immunotherapy Trials Network-09/Keynote-01) found that patients with advanced MCC demonstrated favorable OS and durable tumor control using pembrolizumab compared to historical series using chemotherapy [7]. Given these results, immune therapy is outpacing chemotherapy as standard of care, and both pembrolizumab and avelumab are available for metastatic disease. 

In addition to the metastatic setting where immune therapy is standard of care [7,11], clinical practice in the advanced and adjuvant settings also appears to be shifting towards an immune agent approach. The STAMP trial is evaluating pembrolizumab for patients with completely resected stage I-III MCC [26] and other ongoing phase III studies are evaluating the role for immunotherapy for MCC in the adjuvant (NCT03271372) and advanced disease (NCT03783078) settings. The ADAM protocol (NCT03271372) is evaluating the role for adjuvant avelumab after definitive surgical or radiotherapy in stage III MCC, and KEYNOTE-913 is evaluating the role for pembrolizumab as first line therapy for stage IV MCC (NCT03783078) [27]. Although clinical practice has shifted towards an immune agent approach, a multi-modality approach integrating chemotherapy is still under study. A phase II Australian, TROG 96-07 study examined the role for post-operative synchronous carboplatin (AUC 4.5) and IV etoposide (80 mg/m2) delivered on days 1-3 for 4 cycles concurrent with 50 Gy in 25 fractions for 53 non-metastatic MCC patients with high-risk features. Overall survival, LRC, and distant control were reported to be 76%, 75%, and 76% at 3-years, encouraging further evaluation of chemotherapy with RT for high-risk MCC in future phase III trials [28]. A National Cancer Database (NCDB) study by Chen et al. published in 2015 examined 4,815 patients with head and neck MCC and found that on MVA, post-operative chemo-RT (HR 0.62) and RT (HR 0.80) provided an overall survival benefit over surgery alone with Mohs surgery. Adjuvant CRT appeared to improve OS over adjuvant RT in patients with positive margins (HR 0.48), tumor size at least 3 cm (HR 0.52), and male sex (HR 0.69) [29]. However, we compare these results with caution when explaining the benefit for chemotherapy seen because a primary head and neck MCC may have unique features that differ from MCC in other body sites. In our study, nearly half of the patients in the SEER 18 dataset (44%) had primary head and neck MCC. While chemotherapy was not associated with an improved OS for the head and neck MCC subset, it was also not associated with worse OS either for this subset. Although it is difficult to elucidate precisely why chemotherapy had a neutral effect for this subset, this discrepancy may be due to inherent factors limiting interpretation of a dataset such as SEER. Moreover, chemotherapy was utilized in only 648 cases (13%) out of 5,002 total cases. And because the chemotherapy field is coded either as “yes” or “none/unknown”, the status of the remaining 87% of tumors is indeterminate.

There are additional important limitations to the interpretation of the present study. The SEER database does not report on the specific chemotherapy regimens used, radiation techniques and dosages, completeness of resection or margin status, and adherence to treatment completion. The sequence in relation to chemotherapy, radiation and/or surgery is unknown. The compliance with regimens, and the intent to treat - either definitive or palliative intent for each treatment modality - is unknown. Furthermore, extrapolation of sub-groups may be limited due to small sample sizes. While there was no prognostic association with “black” or “other” race, nor with “uninsured” or “Medicaid” insurance status, these subgroups were small and thus have limited predictive power. Finally, interpretation may be limited due to other confounders and unaccounted variables not identified in our study. Given all these unknown variables, chemotherapy may be a surrogate for more advanced or higher risk disease, and thus the lack of an overall survival benefit with use of chemotherapy for MCC may be due to confounding variables associated with patient selection bias. This was addressed to the best of our ability by looking at each subgroup after IPTW adjustment (Table2, Figure 3). In another SEER study with additional variables, a matched-pairs analysis evaluating 269 patients with MCC found an improved survival with use of irradiation but no cause-specific survival benefit, suggesting a more cautious interpretation of the overall survival benefit endpoint [30].

Despite these drawbacks, our SEER analysis is supported by a robust, large patient cohort with long median follow-up (178 months). The data reflect and updates what has been previously reported including baseline characteristic distributions and risk factors corresponding with worse outcome including male sex, age, and stage. It further elucidates the question of radiation and systemic therapy in the management of this aggressive disease and provides a current snapshot of the characteristics and trends in care for patients with MCC as clinicians shift from a cytotoxic chemotherapy approach to an immunotherapy approach to treatment. Results from ongoing phase III trials evaluating the role for immune-based systemic therapy in the adjuvant and advanced settings are highly anticipated.

## Conclusions

MCC is a rare cutaneous malignancy of neural crest origin with a high rate of metastasis and recurrence. Our analysis provides further evidence supporting the use of radiotherapy. In contrast, there was insufficient evidence to support the use of chemotherapy for MCC - which appears to reflect its ineffective use particularly in the metastatic setting as compared to immune checkpoint inhibitors. Immune therapy has emerged as the standard of care in the setting of metastatic MCC, and ongoing trials in the adjuvant and front-line advanced setting are promising. Future database analyses exploring the impact of immunotherapy as well as the interaction between RT and immunotherapy in MCC are warranted.
